# Effect of eicosapentaenoic acids-rich fish oil supplementation on motor nerve function after eccentric contractions

**DOI:** 10.1186/s12970-017-0176-9

**Published:** 2017-07-12

**Authors:** Eisuke Ochi, Yosuke Tsuchiya, Kenichi Yanagimoto

**Affiliations:** 1 Faculty of Bioscience and Applied Chemistry, Hosei University, 3-7-2, Kajino, Koganei, 184-8584 Japan; 2 Faculty of Modern life, Teikyo Heisei University, Tokyo, Japan; 3 Human Life Science R&D Center, Nippon Suisan Kaisha, Ltd, Tokyo, Japan

**Keywords:** Eicosapentaenoic acid, M-wave, Nerve damage, Muscle damage, Lengthening contractions, Supplement

## Abstract

**Background:**

This study investigated the effect of supplementation with fish oil rich in eicosapentaenoic acid (EPA) and docosahexaenoic acid (DHA) on the M-wave latency of biceps brachii and muscle damage after a single session of maximal elbow flexor eccentric contractions (ECC).

**Methods:**

Twenty-one men were completed the randomized, double-blind, placebo-controlled, and parallel-design study. The subjects were randomly assigned to the fish oil group (*n* = 10) or control group (*n* = 11). The fish oil group consumed eight 300-mg EPA-rich fish oil softgel capsules (containing, in total, 600 mg EPA and 260 mg DHA) per day for 8 weeks before the exercise, and continued this for a further 5 days. The control group consumed an equivalent number of placebo capsules. The subjects performed six sets of ten eccentric contractions of the elbow flexors using a dumbbell set at 40% of their one repetition maximum. M-wave latency was assessed as the time taken from electrical stimulation applied to Erb’s point to the onset of M-wave of the biceps brachii. This was measured before and immediately after exercise, and then after 1, 2, 3, and 5 days. Changes in maximal voluntary isometric contraction (MVC) torque, range of motion (ROM), upper arm circumference, and delayed onset muscle soreness (DOMS) were assessed at the same time points.

**Results:**

Compared with the control group, M-wave latency was significantly shorter in the fish oil group immediately after exercise (*p* = 0.040), MVC torque was significantly higher at 1 day after exercise (*p* = 0.049), ROM was significantly greater at post and 2 days after exercise (post; *p* = 0.006, day 2; *p* = 0.014), and there was significantly less delayed onset muscle soreness at 1 and 2 days after exercise (day 1; *p* = 0.049, day 2; *p* = 0.023).

**Conclusion:**

Eight weeks of EPA and DHA supplementation may play a protective role against motor nerve function and may attenuate muscle damage after eccentric contractions.

**Trial registration:**

This trial was registered on July 14th 2015 (https://upload.umin.ac.jp/cgi-open-bin/ctr/index.cgi).

## Background

It is widely recognized that unaccustomed muscle movement, including eccentric contraction (ECC), causes muscular damage [1, 2]. It has been shown that ECCs result in a loss of muscle strength, delayed onset muscle soreness (DOMS), a limited range of motion (ROM), muscle swelling, increases in serum creatine kinase and myoglobin levels, prolonged transverse relaxation time (T2) on magnetic resonance imaging (MRI), and echo intensity on ultrasound imaging [1, 3, 4]. Interestingly, because these effects differ in the time taken to reach their peak [1, 2], the relationship with each muscle damage marker is not clear. For example, it has been shown that strength loss and limited ROM peak immediately after performing ECCs, whereas DOMS reaches its peak at 1–3 days after the ECCs and T2 and echo intensity, which indicate the distribution of free water and/or interstitial edema, are at their maximum after 3–6 days [1, 2, 4, 5].

Nerve conduction velocity, M-wave latency, and amplitude are often measured to assess motor nerve function [6, 7]. M-wave latency is measured as the time between electrical stimulation and the onset of an M-wave, but this can be influenced by a number of factors, including various processes such as nerve conduction, neuromuscular transmission, and muscle fiber conduction, and sarcolemmal excitability [8]. Previous studies have used M-wave latency to examine nerve disorders such as neuropathy and neural muscular atrophy [6, 9]; these indicated that an increase in M-wave latency reflected the impairment of motor nerves. It has been shown that ECCs caused histological damage in rats, not only in myofibrils, the extracellular matrix, and the triads of the cytoplasmic membrane system [10, 11], but also in nerve fibers and in the thinning of myelin sheaths [12]. Kouzaki et al. [13] reported that M-wave latency delayed by 12% at 24 h and 24% at 48 h after 60 ECCs of the elbow flexors performed by women, suggesting musculocutaneous nerve impairment. However, it is not known whether nutritional strategies have a role for preventing temporal muscular dysfunction after ECCs.

When fish oil is consumed in the diet, the concentration of the long-chain omega-3 polyunsaturated fatty acid increases proportionately in the cellular membranes of muscles [14, 15] as in other organs [16]. In mice, omega-3, especially eicosapentaenoic acid (EPA) and docosahexaenoic acid (DHA), has several important roles in reducing the risk of cardiovascular diseases [17, 18], reducing inflammatory markers [19, 20], and improving vision and cognitive functions including Alzheimer’s disease [21]. Recently, we have shown that EPA and DHA supplementation ameliorated reductions in muscle strength, the development of DOMS, and limited ROM following ECCs [22]. We have hypothesized that, as one of the mechanisms underlying this phenomenon, EPA and DHA play an important role related to nerve structure and/or function. However, as yet no study has investigated the effect of EPA and DHA supplementation on ECC-induced nerve damage.

The aim of the present study was to investigate the effect of 8 weeks of EPA and DHA supplementation on M-wave latency of the biceps brachii after elbow flexion ECCs. We hypothesized that EPA and DHA supplementation for 8 weeks would inhibit the strength loss, limited ROM, DOMS, and muscle swelling after the ECCs and would increase M-wave latency.

## Methods

### Subjects

A total of 21 healthy men (age, 21.0 ± 0.8 years; height, 170.9 ± 5.7 cm; weight, 64.3 ± 6.1 kg; body mass index, 25.0 ± 1.7) were recruited for this study. The sample size was determined by a power analysis (G*power, version 3.0.10, Heinrich-Heine University, Dusseldorf, Germany) by setting the effect size as 1, α level of 0.05 and power (1-β) of 0.80 for the comparison between groups, which showed that at least ten participants were necessary. None had participated in any regular resistance training, restriction of exercise, or other clinical trial, had food allergies to fish, or were taking any supplement or medication. The subjects were requested to avoid interventions such as massage, stretching, and strenuous exercise, and the excessive consumption of food and alcohol, during the experimental period. Prior to participation, they were provided with detailed explanations of the study protocol, and all signed an informed consent form. The study was conducted in accordance with the principles of the Declaration of Helsinki; it was approved by the Ethics Committee for Human Experiments at Juntendo University (ID: 27–66) and has been registered at the University Hospital Medical Information Network Clinical Trials Registry (UMIN-CTR, identifier: UMIN000018285).

### Study design

The study was conducted following the double-blind, placebo-controlled, parallel-group trial design. The subjects were randomly assigned to two groups using a table of random numbers in such a manner as to minimize the inter-group differences in age, body fat, body mass index. According to the previous studies [22, 23], the control group consumed daily placebo capsules for 8 weeks prior to an exercise experiment and for 5 days after the exercise whereas the EPA group consumed EPA supplement capsules as described in the following section. The capsules were taken for a total of 62 days (including the exercise day). The sequence allocation concealment and blinding to subjects and researchers were maintained throughout this period. Compliance to intake was assessed by daily record and a pill count at the end of the study. To help the reliability of the pill count, subjects were given an excess number of pills and asked to return any remaining pills at the end of the study. On the day of exercise testing, M-wave latency, maximal voluntary isometric contraction (MVC) torque, elbow joint ROM, upper arm circumference, and muscle soreness assessed by a visual analog scale, were assessed in the non-dominant arm before exercise. Immediately after these baseline measurements, the subjects performed ECCs with the same arm. All measurements were repeated immediately after the exercise, and then at 1, 2, 3, and 5 days later. Before the subjects started taking the supplement or placebo capsules, we surveyed their nutritional status using the food frequency questionnaire based on food groups (FFQg version 3.5, Kenpakusha, Tokyo, Japan). This was repeated after the 8 weeks supplementation. The primary outcome measures were MVC torque, ROM, M-wave latency, and muscle soreness.

### Supplements

From the previous studies [20, 22, 24] and considering the safety factor [25], the EPA group consumed eight 300-mg EPA-rich fish oil softgel capsules (Nippon Suisan Kaisha Ltd., Tokyo, Japan) per day, a total of 2400 mg per day containing 600 mg EPA and 260 mg DHA. The CON group consumed eight 300 mg corn oil softgel capsules (Nippon Suisan Kaisha Ltd., Tokyo, Japan) per day (not containing EPA and DHA in a total of 2400 mg). The subjects consumed the supplements 30 min after meals with water.

### Eccentric contractions

For the eccentric exercise, the subject sat on a preacher curl bench with his shoulder joint angle at 45° flexion. The dumbbell used was set to weigh 40% of his one repetition maximum arm curl weight. The exercise consisted of six sets of ten maximal voluntary ECCs of the elbow flexors with a rest of 120 s between each set as described in our previous study [22]. The subject was handed the dumbbell in the elbow flexed position (90°) and instructed to lower it to a fully extended position (0°) at an approximately constant speed (30°/s), in time (3 s) with a metronome. The investigator then removed the dumbbell and the subject returned his arm without the dumbbell to the start position for the next eccentric contraction.

### Maximal voluntary isometric contraction torque

For the measurement of MVC torque, the subject performed two 5-s MVCs at a 90° elbow joint angle with a 15-s rest between the contractions. The peak torque of the two contractions was used as the MVC torque. The torque signal was amplified using a strain amplifier (DPM-611B; Kyowa, Tokyo, Japan). The analog torque signal was converted to digital signals with a 16-bit analog-to-digital converter (Power-Lab 16SP; ADInstruments, Bella Vista, Australia). The sampling frequency was set at 2 kHz. The measurement was based on a previous study [26].

### Range of motion of the elbow joint

To examine elbow joint ROM, two elbow joint angles (extended and flexed) were measured using a goniometer (Takase Medical, Tokyo, Japan). The extended joint angle was recorded while the subject attempted to fully extend the joint with the elbow held by his side and the hand in supination [22] The flexed joint angle was determined while the subject attempted to fully flex the joint from an equally fully extended position with the hand supinated. The ROM was calculated by subtracting the flexed joint angle from the extended joint angle.

### Upper arm circumference

The upper arm circumference was measured at 3, 5, 7, 9, and 11 cm above the elbow joint using a Gulick tape measure while the subject stood with his arm relaxed by his side. The mean value of five measurements was used for the analysis. Measurement marks were maintained throughout the experimental period using a semi-permanent ink marker, and a well-trained investigator made the measurements [22]. The mean value of measurements was used for analysis.

### Ultrasonography

B-mode ultrasound pictures of the upper arm were taken of the biceps brachii using ultrasound (Aixplorer, SuperSonic, France), with the probe placed 9 cm from the elbow joint at the position marked for the circumference measurement. The gains and contrast were kept consistent over the experimental period. The transverse images were transferred to a computer as bitmap (.bmp) files and analyzed by a computer. The cross-sectional area of the elbow flexors was determined by measuring the distance between the subcutaneous fat layer and the edge of the humerus [27] on the transverse images of the biceps brachii. The average echo intensity for the region of interest (20 × 20 mm) was calculated by the computer image analysis software that provided a gray scale histogram (0, black; 100, white) for the region as described in previous study [27].

### Muscle soreness

Muscle soreness in the elbow flexors was assessed using a 10-cm visual analog scale in which 0 indicated “no pain” and 10 was the “the worst pain imaginable” [4]. The subject relaxed with his arm in a natural position; the investigator then palpated the upper arm using a thumb and the subject indicated his pain level on the visual scale. All tests were conducted by the same investigator, who had been trained to apply the same pressure over time and between subjects.

### M-wave latency

The musculocutaneous nerve was stimulated (for a pulse duration of 10 ms) by a bipolar surface electrode with an inter-electrode distance of 23 mm and a diameter of 0.8 mm (NM-420S, Nihon Kohden, Tokyo, Japan). The stimulation electrode was placed over Erb’s point located on the supraclavicular fossa and sternocleidomastoid muscle and connected to an electrical stimulator (DS7AH, Digitimer Ltd., Welwyn Garden City, UK). The M-wave latency and amplitude were assessed according to the instructions in the “Manual of Nerve Conduction Studies” [28] and as described in our previous study [13]. The subject sat on a chair with both arms at his side and placed his forearm on his lap, resulting in the elbow joint being at approximately 10° flexion. This position was kept consistent between measures, and a plastic goniometer was used to reproduce the elbow joint angle. A monopolar surface electrode was placed at the mid-belly of the biceps brachii long head, a reference electrode was placed proximal to the antecubital fossa in the region of the junction of the muscle fibers and the biceps tendon, and a ground electrode was placed on the acromion [13]. The electrode locations were marked with a semi-permanent marker to ensure the same placement over time. The stimulation current was gradually increased to obtain the maximal M-wave (final stimulation current: 14–18 mA), and it was confirmed that a further increase in the current did not increase the M-wave amplitude. Data acquisition and analysis were performed using Lab Chart 7 (ADInstruments, Bella Vista, Australia). The relative increase in the M-wave latency from the pre-ECC value was then calculated. This measurement was based on a previous study [13].

### Statistical analyses

All analyses were performed using SPSS Statistics software version 22.0 (IBM Corp., Armonk, NY). Values are expressed as means ± standard deviation (SD). MVC torque, ROM, echo intensity, circumference, CSA, and nerve conduction latency values at post, day1, day2, day3, and day5 were calculated by relative changes from baseline (100%). MVC, ROM, upper arm circumference, echo intensity, cross-sectional area, muscle soreness, and M-wave latency over time were compared between the CON and EPA groups by two-way repeated-measure analysis of variance (ANOVA). When a significant main effect or interaction was found, Bonferroni’s correction was performed for the post-hoc testing. A *p*-value of <0.05 was considered statistically significant.

## Results

### Physical characteristics and nutritional status

No subject dropped out during the study period. No significant differences were observed between the EPA group and the CON group for age, weight, and body mass index (EPA; *n* = 10; age, 20.7 ± 0.7 years; height, 171.5 ± 6.6 cm; weight, 63.0 ± 6.3 kg; body mass index, 25.0 ± 2.0, CON; *n* = 11; age, 21.3 ± 0.9 years; height, 170.7 ± 5.0 cm; weight, 65.5 ± 5.9 kg; body mass index, 25.0 ± 1.5). The food frequency questionnaire results showed no significant difference in nutritional status between the EPA group (energy, 2578.1 ± 359.4 kcal; protein, 88.7 ± 18.7 g; fat, 91.9 ± 18.5 g; carbohydrate, 330.4 ± 60.0 g; omega-3 fatty acid, 2.4 ± 0.7 g) and the CON group (energy, 2273.4 ± 607.7 kcal; protein, 80.7 ± 20.7 g; fat, 92.9 ± 25.0 g; carbohydrate, 268.6 ± 90.8 g; omega-3 fatty acid, 2.3 ± 0.6 g) before the intake of supplements (Table 1). These parameters did not change during the experimental period. Blood analyses showed that EPA was significantly higher in the EPA group than in the CON group after 8 weeks intake of supplements﻿,﻿ whereas DHA was no significantly difference between two groups (data not shown).

**Table 1:** Physical characteristics of subjects

	Age (year)	Height (cm)	Weight (kg)	BMI
CON (*n* = 11)	21.3 ± 0.9	170.7 ± 5.0	65.5 ± 5.9	25.0 ± 1.5
EPA (*n* = 10)	20.7 ± 0.7	171.5 ± 6.6	63.0 ± 6.3	25.0 ± 2.0

n.s.

### Maximal voluntary isometric contraction torque

**Fig. 1:**
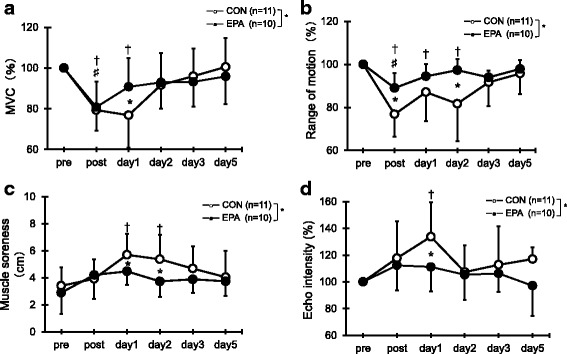
Changes (mean ± SD) of maximal voluntary isometric contraction (MVC) torque (**a**), range of motion (ROM) (**b**), muscle soreness (**c**), and echo intensity (**d**) measured before (pre) and immediately after (post) the eccentric contractions exercise and then 1, 2, 3, and 5 days later in the control and EPA groups. The values of MVC torque, ROM, and echo intensity at post, 1, 2, 3, and 5 days after eccentric contractions were calculated by relative changes from baseline (100%). * *p* < 0.05 for the difference between groups; † *p* < 0.05 for the difference from the pre-exercise value in the control group, # *p* < 0.05 for the difference from pre-exercise value in the EPA group

Compared with the pre-exercise value, MVC torque in the CON group had significantly decreased immediately after the exercise and 1 day later (post; *p* = 0.001, day 1; *p* = 0.003; Fig. 1a). MVC torque in the EPA group decreased immediately after the exercise compared with the pre-exercise level (*p* = 0.010). MVC was significantly higher in the EPA group than in the CON group at 1 day after the exercise (CON 76.8% ± 16.0%, EPA 90.5% ± 14.0%, *p* = 0.049). The results for the absolute MVC were similar to these.

### Range of elbow motion

As shown in Fig. 1b, a significant decrease in ROM was observed in the CON group immediately after the exercise (a reduction of 23.2%, *p* = 0.000), and ROM continued to be lower than baseline at 1 and 2 days (day 1; *p* = 0.003, day 2; *p* = 0.012). ROM in the EPA group decreased immediately after the exercise compared with the pre-exercise level (a reduction of 12.0%, *p* = 0.013). ROM was significantly greater in the EPA group than in the CON group immediately after exercise and 2 days later (post: CON 76.8% ± 10.4%, EPA 88.1% ± 7.2%, *p* = 0.006; day 2: CON 81.7% ± 17.4%, EPA 96.2% ± 6.3%, *p* = 0.014).

### Muscle soreness

Compared with the pre-exercise value, a significant level of muscle soreness was indicated by the CON group using the visual analog scale at 1 and 2 days after exercise (day 1; *p* = 0.006, day 2; *p* = 0.021; Fig 1c). In contrast, no increase in muscle soreness was indicated by the EPA group at any time points. Significantly greater muscle soreness was observed in the CON group compared with the EPA group at 1 day (CON, 5.7 ± 1.6 cm vs. EPA, 4.5 ± 1.0 cm; *p* = 0.049) and 2 days (CON, 5.4 ± 1.8 cm vs. EPA, 3.7 ± 1.2 cm; *p* = 0.023) after the exercise.

### Echo intensity

In the CON group, echo intensity increased at 1 day after exercise (133.8% ± 25.9%, *p* = 0.005; Fig. 1d), but there was no significant increase in echo intensity in the EPA group at any time point. The echo intensity was significantly higher for the CON group than the EPA group at 1 day after the exercise (CON, 133.8 ± 25.9% vs. EPA, 111.2 ± 18.2%; *p* = 0.045).

### Upper arm circumference and cross-sectional area of the flexors

**Fig. 2:**
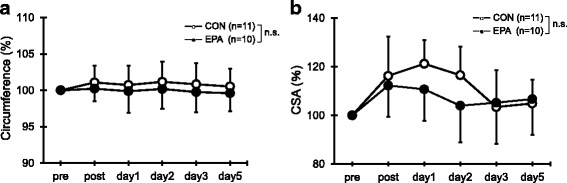
Changes (mean ± SD) of upper arm circumference (**a**) and cross-sectional area (CSA) of the elbow flexors (**b**) measured before (pre) and immediately after (post) the eccentric contractions exercise and then 1, 2, 3, and 5 days later in the control and EPA groups. The values of upper arm circumference and CSA at post, 1, 2, 3, and 5 days after eccentric contractions were calculated by relative changes from baseline (100%). n.s. not significant

No significant difference in the upper arm circumference from the pre-exercise values or between the two groups was observed at any time point (Fig. 2a). Similarly, there was no significant difference in the cross-sectional area of the elbow flexors at any time point (Fig. 2b).

### M-wave latency

**Fig. 3:**
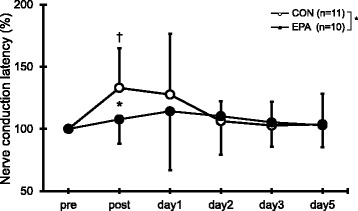
Changes (mean ± SD) of nerve conduction latency measured before (pre) and immediately after (post) the eccentric contractions exercise and then 1, 2, 3, and 5 days later in the control and EPA groups. The values of nerve conduction latency at post, 1, 2, 3, and 5 days after eccentric contractions were calculated by relative changes from baseline (100%). * *p* < 0.05 for the difference between groups, † *p* < 0.05 for the difference from the pre-exercise value in the control group

In the CON group, but not the EPA group, M-wave latency increased immediately after the exercise (*p* = 0.010; Fig. 3). In addition, M-wave latency was significantly longer in the CON group than in the EPA group immediately after the exercise (CON, 133.0 ± 32.1% vs. EPA, 113.3 ± 26.5%; *p* = 0.040).

## Discussion

This study investigated the effect of EPA and DHA supplementation on the M-wave latency of the biceps brachii and on muscle damage after single session of maximal elbow flexion ECC exercises. The results demonstrated that EPA and DHA supplementation inhibited the loss of muscle strength, limitation of ROM, development of DOMS, and increases in echo intensity and M-wave latency. These results support our original hypothesis.

The reduction of isometric torque was significantly inhibited in the EPA group. Our recent study also showed that 600 mg EPA and 260 mg DHA supplementation for 8 weeks inhibited strength loss following elbow flexion ECC exercises [22]. To the best of our knowledge, that study was the first to demonstrate that EPA and DHA supplementation had a positive effect on MVC torque following ECC exercises, and the present study supports those results. Strength loss following ECCs has been considered to be the result of disruption of the myofibrils, muscle membranes, and neuromuscular junctions, and abnormal calcium (Ca^2+^) levels [3]. Temporary strength loss following ECCs has also been assumed to be due to excitation–contraction coupling failure [29]. Importantly, omega-3 polyunsaturated fatty acids are a major component of the cell membrane, with the total concentration in muscle cellular membrane significantly increasing after the ingestion of fish oil [30]. Thus, it is possible that the muscle membrane structure may be protected by EPA supplementation and that EPA and DHA thereby reduced the muscular dysfunction that resulted from the ECCs at day 1.

Nerve injuries are classified as neurapraxia, axonotmesis, or neurotmesis [31–33]. In neurapraxia, nerve conduction is impaired by a segmental myelination of the nerve trunk without axonal degeneration. The causes of neurapraxia include mechanically induced lesions, ischemia, inflammation, toxic compounds, and metabolic disturbances [31, 34], and M-wave latency increases without an apparent change in M-wave amplitude [33]. In the present study, the M-wave amplitude did not significantly change after the ECCs (data not shown), and the prolonged M-wave latency was not observed at 2 days post-exercise. Thus, the effect of ECCs on the nerve may have been similar to neurapraxia. The present study also demonstrated that EPA and DHA supplementation inhibited the increased M-wave latency following the eccentric exercise. We believe that this is the first report to show that EPA and DHA have important roles in counteracting the muscle dysfunction resulting from ECCs. Braddom et al. [35] observed structural damage to musculoskeletal nerves and ischemic neuropathy with high-intensity weight lifting exercise. In addition, it has been shown in rats that repeated sessions of ECCs caused histological damage in nerve fibers and thinning of myelin sheaths [12]. It is therefore possible that the decreased M-wave latency was associated with a protective role for EPA and DHA with regard to nerve fiber damage and myelin sheaths thinning. As mentioned earlier, one cause of neurapraxia is mechanically induced inflammation [31, 34]; we assume that EPA and DHA reduce the inflammation resulting from nerve injury.

Our study also demonstrated that EPA and DHA supplementation had a preventive effect on ROM and DOMS following eccentric exercise. These results are consistent with those of previous studies [22, 24]. Tartibian et al. [24] showed that daily supplementation with 324 mg EPA and 216 mg DHA attenuated the limitation of ROM after 40 min of bench stepping. Similarly, our recent study demonstrated that 600 mg EPA and 260 mg DHA supplementation for 8 weeks inhibited the limitation of ROM and the development of DOMS [22]. The limited ROM following ECC has been attributed to an inflammatory response within myofibrils leading to an increase in passive stiffness [36]. Although DOMS can be attributed to a combination of several factors, previous studies have suggested that its primary cause is a local inflammatory response [11, 37]. It is well established that EPA and DHA have anti-inflammatory effects that reduce levels of interleukin-6 [22]; we therefore suggest that their inhibition of limited ROM and the development of DOMS could be attributed to their anti-inflammatory effects.

In this study, the echo intensity was significantly lower in the EPA group than in the CON group 1 day after the ECC exercise. Increased echo intensity is associated with edema of the muscle due to trauma, ischemia, or infraction [1]. Indeed, previous studies have confirmed that echo intensity in the biceps brachii and brachialis increases with elbow flexors after ECC exercise [1, 26, 38]. In addition, because echo intensity appears to be related to creatine kinase level after ECC [1], it has the potential to be a useful marker of muscle damage. Although the mechanism is unclear, the results in present study suggest that EPA and DHA supplementation may inhibit edema. However, no significant difference was observed in the upper arm circumference. This observation is similar to that of our previous study [22], and we assume that it related to a limitation of the study method. Although we used a Gulick tape measure, we could not exclude the effects of other muscles, fat, skin, etc. For this reason, in the present study we also calculated the cross-sectional area using ultrasonography. However, we observed no significant difference between groups, although the cross-sectional area showed a non-significant tendency to be smaller in the EPA group than in the CON group. The reason for this could be that the intensity of ECC and the reduction of muscle strength were lower than in the previous studies [2], and that the accuracy of ultrasonography is lower than that of MRI. A further study is needed that uses MRI to determine the response after severe ECC exercise.

The present study had the following three limitations. First, we did not evaluate the inflammatory response such as C-reactive protein (CRP) and tumor necrosis factor-alpha (TNF-α), and interleukin (IL)-6 [22, 39]. Although the results demonstrated that EPA and DHA supplementation had a positive effect on DOMS and ROM following eccentric exercise, the actual inflammatory response was unknown. Second, we investigated only a single dose of EPA and DHA administration. It has been shown that the amount of EPA and DHA is limited 3000 mg in total per 1 day for the safety in human by natural medicines comprehensive database [25]. Regarding the effect of EPA and DHA supplementation on muscle damage, the minimal effect was with 540 mg/day (EPA and DHA) according to Tartibian et al [24]. Hence, we have decided to use the amount of supplementation (600 mg EPA and 260 mg DHA). However, further investigation is needed to study the different doses of EPA and DHA to elucidate the appropriate amount. Third, we used one bout of eccentric exercise model, which was a single-exercise, to untrained subjects. Therefore, our observations could not provide to athletes and multiple training sessions. Further study is required to investigate this point.

## Conclusions

In summary, we showed that 600 mg EPA and 260 mg DHA supplementation for 8 weeks inhibited the increased M-wave latency following a session of intense ECC exercise, as well as the strength loss, limitation of ROM, and development of DOMS. We speculate that the mechanism underlying these observations may be related to the improvement of nerve structure and function. These findings of the beneficial effects of EPA and DHA supplementation are of importance for applied sport scientists, nutritionists, and strength and conditioning professionals and could help them to design better nutritional interventions aimed at preventing temporary muscle strength loss, limited flexibility, and DOMS after exercise.

## References

[1] Nosaka K, Clarkson PM (1996). Changes in indicators of inflammation after eccentric exercise of the elbow flexors. Med Sci Sports Exerc.

[2] Ochi E, Tsuchiya Y, Nosaka K (2016). Differences in post-exercise t2 relaxation time changes between eccentric and concentric contractions of the elbow flexors. Eur J Appl Physiol.

[3] Clarkson PM, Sayers SP (1999). Etiology of exercise-induced muscle damage. Can J Appl Physiol.

[4] Tsuchiya Y, Kikuchi N, Shirato M (2015). Differences of activation pattern and damage in elbow flexor muscle after isokinetic eccentric contractions. Isokinet Exerc Sci.

[5] Larsen RG, Ringgaard S, Overgaard K (2007). Localization and quantification of muscle damage by magnetic resonance imaging following step exercise in young women. Scand J Med Sci Sports.

[6] Colak T, Bamac B, Alemdar M (2009). Nerve conduction studies of the axillary, musculocutaneous and radial nerves in elite ice hockey players. J Sports Med Phys Fitness.

[7] Maccabee PJ, Eberle LP, Stein IA (2011). Upper leg conduction time distinguishes demyelinating neuropathies. Muscle Nerve.

[8] Mallik A, Weir AI (2005). Nerve conduction studies: essentials and pitfalls in practice. J Neurol Neurosurg Psychiatry.

[9] Kaplan PE (1976). Sensory and motor residual latency measurements in healthy patients and patients with neuropathy-part 1. J Neurol Neurosurg Psychiatry.

[10] Piitulainen H, Komi P, Linnamo V (2008). Sarcolemmal excitability as investigated with m-waves after eccentric exercise in humans. J Electromyogr Kinesiol.

[11] Proske U, Morgan DL (2001). Muscle damage from eccentric exercise: mechanism, mechanical signs, adaptation and clinical applications. J Physiol.

[12] Kouzaki K, Kobayashi M, Nakamura KI (2016). Repeated bouts of fast eccentric contraction produce sciatic nerve damage in rats. Muscle Nerve.

[13] Kouzaki K, Nosaka K, Ochi E (2016). Increases in m-wave latency of biceps brachii after elbow flexor eccentric contractions in women. Eur J Appl Physiol.

[14] Andersson A, Nalsen C, Tengblad S (2002). Fatty acid composition of skeletal muscle reflects dietary fat composition in humans. Am J Clin Nutr.

[15] Henry R, Peoples GE, McLennan PL (2015). Muscle fatigue resistance in the rat hindlimb in vivo from low dietary intakes of tuna fish oil that selectively increase phospholipid n-3 docosahexaenoic acid according to muscle fibre type. Br J Nutr.

[16] Charnock JS, Abeywardena MY, Poletti VM (1992). Differences in fatty acid composition of various tissues of the marmoset monkey (*Callithrix jacchus*) after different lipid supplemented diets. Comp Biochem Physiol Comp Physiol.

[17] Pauwels EK, Kostkiewicz M (2008). Fatty acid facts, part iii: cardiovascular disease, or, a fish diet is not fishy. Drug News Perspect.

[18] Yokoyama M, Origasa H, Matsuzaki M (2007). Effects of eicosapentaenoic acid on major coronary events in hypercholesterolaemic patients (jelis): a randomised open-label, blinded endpoint analysis. Lancet (London, England).

[19] Kelley DS, Siegel D, Fedor DM (2009). DHA supplementation decreases serum c-reactive protein and other markers of inflammation in hypertriglyceridemic men. J Nutr.

[20] Tartibian B, Maleki BH, Abbasi A (2011). Omega-3 fatty acids supplementation attenuates inflammatory markers after eccentric exercise in untrained men. Clin J Sport Med.

[21] Lim GP, Calon F, Morihara T (2005). A diet enriched with the omega-3 fatty acid docosahexaenoic acid reduces amyloid burden in an aged alzheimer mouse model. J Neurosci.

[22] Tsuchiya Y, Yanagimoto K, Nakazato K (2016). Eicosapentaenoic and docosahexaenoic acids-rich fish oil supplementation attenuates strength loss and limited joint range of motion after eccentric contractions: a randomized, double-blind, placebo-controlled, parallel-group trial. Eur J Appl Physiol.

[23] Metcalf RG, James MJ, Gibson RA (2007). Effects of fish-oil supplementation on myocardial fatty acids in humans. Am J Clin Nutr.

[24] Tartibian B, Maleki BH, Abbasi A (2009). The effects of ingestion of omega-3 fatty acids on perceived pain and external symptoms of delayed onset muscle soreness in untrained men. Clin J Sport Med.

[25] Administration. UFaD: Letter regarding dietary supplement health claim for omega-3 fatty acids and coro- nary heart disease. Docket No 91 N-0103. 2000.

[26] Chen TC, Lin KY, Chen HL (2011). Comparison in eccentric exercise-induced muscle damage among four limb muscles. Eur J Appl Physiol.

[27] Chen TC, Nosaka K (2006). Responses of elbow flexors to two strenuous eccentric exercise bouts separated by three days. J Strength Cond Res.

[28] Buschbacher R (2000). Manual of nerve conduction studies.

[29] Warren GL, Lowe DA, Hayes DA (1993). Excitation failure in eccentric contraction-induced injury of mouse soleus muscle. J Physiol.

[30] Helge JW, Therkildsen KJ, Jorgensen TB (2001). Eccentric contractions affect muscle membrane phospholipid fatty acid composition in rats. Exp Physiol.

[31] Campbell WW (2008). Evaluation and management of peripheral nerve injury. Clin Neurophysiol.

[32] Martins RS, Bastos D, Siqueira MG (2013). Traumatic injuries of peripheral nerves: a review with emphasis on surgical indication. Arq Neuropsiquiatr.

[33] Seddon HJ (1942). A classification of nerve injuries. Br Med J.

[34] Menorca RM, Fussell TS, Elfar JC (2013). Nerve physiology: mechanisms of injury and recovery. Hand Clin.

[35] Braddom RL, Wolfe C (1978). Musculocutaneous nerve injury after heavy exercise. Arch Phys Med Rehabil.

[36] Chleboun GS, Howell JN, Conatser RR (1998). Relationship between muscle swelling and stiffness after eccentric exercise. Med Sci Sports Exerc.

[37] Clarkson PM, Nosaka K, Braun B (1992). Muscle function after exercise-induced muscle damage and rapid adaptation. Med Sci Sports Exerc.

[38] Chen HL, Nosaka K, Chen TC (2012). Muscle damage protection by low-intensity eccentric contractions remains for 2 weeks but not 3 weeks. Eur J Appl Physiol.

[39] Bloomer RJ, Larson DE, Fisher-Wellman KH (2009). Effect of eicosapentaenoic and docosahexaenoic acid on resting and exercise-induced inflammatory and oxidative stress biomarkers: a randomized, placebo controlled, cross-over study. Lipids Health Dis.

